# Characterization of antibody response in asymptomatic and symptomatic SARS-CoV-2 infection

**DOI:** 10.1371/journal.pone.0253977

**Published:** 2021-07-02

**Authors:** Serena Marchi, Simonetta Viviani, Edmond J. Remarque, Antonella Ruello, Emilio Bombardieri, Valentina Bollati, Gregorio P. Milani, Alessandro Manenti, Giulia Lapini, Annunziata Rebuffat, Emanuele Montomoli, Claudia M. Trombetta

**Affiliations:** 1 Department of Molecular and Developmental Medicine, University of Siena, Siena, Italy; 2 Department of Virology, Biomedical Primate Research Centre, Rijswijk, The Netherlands; 3 Humanitas Gavazzeni, Bergamo, Italy; 4 Department of Clinical Sciences and Community Health, University of Milan, Milan, Italy; 5 Pediatric Unit, Fondazione IRCCS Ca’ Granda Ospedale Maggiore Policlinico, Milan, Italy; 6 VisMederi srl, Siena, Italy; 7 VisMederi Research srl, Siena, Italy; 8 Presidio Ospedaliero di Campostaggia, Località Campostaggia, Poggibonsi, Italy; University of South Carolina, UNITED STATES

## Abstract

SARS-CoV-2 pandemic is causing high morbidity and mortality burden worldwide with unprecedented strain on health care systems. To investigate the time course of the antibody response in relation to the outcome we performed a study in hospitalized COVID-19 patients. As comparison we also investigated the time course of the antibody response in SARS-CoV-2 asymptomatic subjects. Study results show that patients produce a strong antibody response to SARS-CoV-2 with high correlation between different viral antigens (spike protein and nucleoprotein) and among antibody classes (IgA, IgG, and IgM and neutralizing antibodies). The antibody peak is reached by 3 weeks from hospital admission followed by a sharp decrease. No difference was observed in any parameter of the antibody classes, including neutralizing antibodies, between subjects who recovered or with fatal outcome. Only few asymptomatic subjects developed antibodies at detectable levels.

## Introduction

On March 11, 2020, the World Health Organization (WHO) Director General declared a pandemic situation due to a novel coronavirus causing a Severe Acute Respiratory Syndrome (SARS) rapidly spreading worldwide [1]. The novel coronavirus (CoV) SARS-CoV-2 has been firstly identified in Wuhan, Hubei Province, China, at the end of 2019 when a cluster of atypical pneumonia occurred [1, 2]. In January 2020, SARS-CoV-2 was isolated and sequenced as a CoV genetically related to the highly pathogenic CoV (SARS-CoV-1) responsible for the 2003 SARS epidemic that spread mainly in Asia with approximately 10% case fatality rate (CFR) [3]. Since 2004 SARS-CoV-1 circulation in humans ended whereas a third highly pathogenic CoV emerged in 2012 in Saudi Arabia causing the Middle East Respiratory Syndrome (MERS) [2, 4–7]. Since then MERS-CoV has spread to 27 countries with limited human-to-human transmission, and a CFR of approximately 34.4%, according to the most recent WHO report [7]. As SARS-CoV-1 and MERS-CoV, the SARS-CoV-2 virus is an enveloped, single-stranded, and positive-sense RNA virus belonging to the Betacoronavirus Genus, *Coronaviridae* family. SARS-CoV-2 genome, as the other emerging pathogenic human CoVs, encodes four major structural proteins: spike (S), envelope (E), membrane (M), nucleocapsid (N); approximately 16 nonstructural proteins (nsp1–16), and five to eight accessory proteins. Among them, the S protein plays an essential role in viral attachment, fusion, entry, and transmission. The S protein is the common target antigen for antibodies and vaccine development [8–11]. After SARS-CoV-2 infection, different categories of antibodies are circulating in serum as Immunoglobulin G (IgG), Immunoglobulin M (IgM) and Immunoglobulin A (IgA) mainly targeting two viral proteins, the S protein and the nucleoprotein (NP). The latter is abundant and highly expressed however, due to its biological function, it seems to be unlikely that antibodies against NP have neutralizing activity. The S protein contains the receptor binding domain (RBD), which mediates the binding to the host cell through the human Angiotensin-Converting Enzyme 2 (ACE2) and the fusion of viral and cellular membranes. Based on SARS-CoV-2 evidence as well as for other CoVs, the S protein seems to be the main target for neutralizing antibodies [12–14]. In COVID-19 patients, the levels of IgM and IgG increased at least 10 days after the onset of symptoms, most patients showed seroconversion within the first 3 weeks and the median time to seroconversion was 20 days [15, 16]. IgG and IgM seroconversion can occur simultaneously or sequentially [17] while IgA timing seems to be the most variable [15–18]. Common serological tests used are ELISA‐based with different combinations of coatings on the S protein (S1, S1+S2, S1‐S2, extracellular domain, RBD). The NP-based ELISA- is also used [19]. ELISAs have some advantages, such as high readout, speed of testing, and a BSL2 laboratory [20] (24). However, the Virus‐Neutralization assay (VN) is currently considered the gold‐standard as capable of measuring neutralizing antibodies that mimic in vitro the in vivo functional activity of blocking the virus [21].

SARS-CoV-2 predominant way of transmission is human-to-human through respiratory droplets, however, close contact with infected surfaces or objects may also be an occasional way of transmission as the virus is excreted and detectable in saliva and stool [6, 9, 22]. SARS-CoV-2 disease, or COVID-19, ranges from a mild upper/lower respiratory tract infection that resolves in a few days without sequelae to more serious disease with fever, cough, shortness of breath, myalgias, fatigue, confusion, headache, sore throat, acute respiratory distress syndrome, leading to respiratory or multiorgan failure [6, 9, 22]. The fatality rate is high in people with underlying comorbidities, such as diabetes, hypertension, chronic respiratory disease, or cardiovascular disease and in the elderly [9, 22]. Almost one year after the first COVID-19 cases were reported in Wuhan, as of 13 December 2020, there have been over 70 million cases and over 1.5 million deaths reported to WHO [23] with Europe being one of the most affected areas. COVID-19 pandemic is causing high morbidity and mortality burden worldwide, an unprecedented strain on health care systems, and social and economic disruption [24].

Italy has been affected by COVID-19 as early as February 2020 with the first SARS-CoV-2 case identified in Codogno at the end of February 2020, considered as the Italian index case. However, some evidence has later emerged that the virus had been circulating in Italy and Europe since autumn 2019 [25–28]. Italy suffered the first epidemic wave from February 2020 until June 2020 when the whole country was under strict lockdown. The most affected areas were in the Northern and, to a less extent, in Central Italy, while the Southern part of the country was relatively unaffected [29, 30]. During the summer COVID-19 remained endemic, with a second epidemic wave starting in October 2020 that led to a subsequent nationwide lockdown in November 2020. As of the 13^th^ of December 2020, more than 1.8 million confirmed cases and more than 64.000 deaths due to SARS-CoV-2 were reported to ISS (Istituto Superiore di Sanità), Rome [29]. The mean age of fatalities from COVID-19 was 80 years, 42,4% were women and more than 90% had one or more co-morbidity as ischemic heart disease, diabetes, active cancer, atrial fibrillation, dementia, and a history of stroke [31].

The emerging and rapid diffusion of COVID-19 has risen the calls for more targeted research in the field [32] helping to elucidate the mechanism of infection, protection, or rapid evolution until fatal outcome. We present here a study performed in hospitalized COVID-19 patients to investigate the time course of the antibody response in relation to the outcome, and as explorative comparison, to investigate the time course of the antibody response in SARS-CoV-2 asymptomatic subjects.

## Material and methods

### Study population

This was a retrospective study on COVID-19 patients and SARS-CoV-2 asymptomatic subjects collected between March and May 2020 during the first epidemic wave occurred in Italy.

A total of 42 COVID-19 patients, hospitalized at Humanitas Gavazzeni (Bergamo, Italy), were retrospectively selected for this study, of whom 35 (22 males and 13 females) recovered and 7 (3 males and 4 females) had a fatal outcome. All subjects were admitted to hospital with a diagnosis of interstitial pneumonia confirmed by chest radiograph or a CAT (computerized axial tomography) and had rhino-pharyngeal swab positive to SARS-CoV-2 (Real-Time PCR Thermo Fisher Scientific). Six (6) patients required care in the intensive care unit (ICU), the others were hospitalized in the general medicine unit. Out of 7 deceased patients, 3 were hospitalized in ICU and 4 in the general medicine unit.

Serum samples were collected at different time points from March to April 2020 for diagnostic/therapeutic purposes. We selected patients who had at least 5 blood samples available during the period of hospital stay (baseline, day 2, day 6, day 12–14, day 18–20, day 27–30). Demographic and clinical variables reported in this study were those collected at hospital admission. For the purpose of this study patients were categorized according to the outcome: recovered or deceased.

During the first phase of the COVID-19 epidemic, little was known about this novel CoV and there was no standard therapy, so the management changed over time. The Italian Society of Infectious and Tropical Diseases recommended as therapy hydroxychloroquine, antiviral agents, steroids, low molecular weight heparin and oxygen support in different combinations according to the clinician’s evaluation. The antibiotic therapy was adopted only in case of suspected or confirmed bacterial superinfection.

Serum samples from 25 asymptomatic subjects who presented a positive rhino-pharyngeal swab for SARS-CoV-2 were collected as part of the UNICORN project and were analysed in the present study [33].

This study was approved by the Ethics Committee of the University of Siena (approval number 17373, approval date June 1, 2020), by the Ethics Committee of Humanitas Gavazzeni (approval number 236, approval date September 22, 2020 Protocol 670/20). All serum samples have been fully anonymized before testing. The UNICORN study was approved by the Ethics Committee of the University of Milan (approval number 17/20, approval date March 6, 2020). All participants signed an informed consent form.

### Serological assay

#### ELISA

All serum samples were tested by commercial ELISA for the detection of IgA, IgG, and IgM against the S1 of SARS-CoV-2 (Aeskulisa^®^ SARS-CoV-2 S1 IgA, IgM, IgG, Aesku. Diagnostics, Wendelsheim, Germany) and for the detection of IgG against the NP of SARS-CoV-2 (Aeskulisa^®^ SARS-CoV-2 NP IgG, Aesku.Diagnostics, Wendelsheim, Germany).

According to the manufacturer’s instructions, quantitative analysis was performed by use of a 4-parameter logistic standard curve obtained by plotting the optical density (OD) values measured for 4 calibrators against their antibody activity (U/ml) using logarithmic/linear coordinates. Antibody activities of the samples were evaluated from OD values using the generated curve and considered positive if >12 U/ml.

#### Virus neutralization assay

The virus neutralization (VN) assay has been performed as previously reported [20]. Briefly, serum samples were heat-inactivated for 30 minutes at 56°C and, starting from 1:10 dilution, were mixed with an equal volume of SARS-CoV-2 (2019‐nCov/Italy‐INMI1 strain) viral solution containing 100 Tissue Culture Infective Dose 50% (TCID50). After 1 hour of incubation at room temperature, 100μl of virus-serum mixture were added to a 96-well plate containing VERO E6 cells with 80% confluency. Plates were incubated for 3 days at 37°C, 5% CO_2_ in humidified atmosphere, then inspected for presence/absence of cytopathic effect (CPE) by means of an inverted optical microscope. A CPE higher than 50% indicated infection. The VN titer was expressed as the reciprocal of the highest serum dilution showing protection from viral infection and CPE.

### Statistical analysis

All statistical analyses were performed using Microsoft R-Open version 3.5.0 (R Core Team (2018). R: A language and environment for statistical computing. R Foundation for Statistical Computing, Vienna, Austria. URL https://www.R-project.org/). For patient baseline characteristics continuous variables were evaluated using Mann-Whitney tests and for categorical variables Chi-square tests were used. Seroconversion rates were compared using Fisher’s exact test. Antibody levels were statistically evaluated using t-tests. Statistical significance was set at p<0.05, two tailed.

## Results

### COVID-19 patients

Between March and April 2020, a total of 42 subjects were retrospectively selected, of whom 35 recovered and 7 had a fatal outcome. The median age at admission was 64.0 years (interquartile range (IQR) 56.0–71.5) for those who recovered and 69.0 years (IQR 64.5–72.0) for deceased patients. The median length of stay in the hospital was similar in both groups with 11.0 days (IQR 9.0–24.5) and 10.0 (IQR 6.0–15.59) for recovered and deceased patients, respectively. The mean number of pre-existing conditions in recovered and deceased was 1.53 (standard deviation (SD) 1.25) and 2.0 (SD 1.41), respectively, and comorbidities were indicated in 1.88 (SD 1.36) and 3.5 (SD 3.54) of recovered and deceased patients, respectively. Forty-five per cent (45%) of patients had at least one comorbidity. Main co-morbidities were cardiovascular disease, diabetes, cerebrovascular disease, hypertension and COPD (Chronic Obstructive Pulmonary Disease). No differences were found between recovered and deceased patients when compared for symptoms at admission (fever, cough, diarrhea, dyspnea) or presence/absence of comorbidities and/or preexisting conditions. The other demographic, clinical, and blood chemistry variables collected at baseline were similar between the two groups, with exception of ALT that showed to be statistically significantly higher (p-value 0.021) in subjects who recovered (Table 1).

**Table 1:** Baseline characteristics of COVID-19 patients according to outcome. Median (IQR).

*Parameter*	*Recovered*	*Deceased*	*P-value*
Sex	22 M / 13 F	3 M / 4 F	0.574
Age	64.0 (56.0 to 71.5)	69.0 (64.5 to 72.0)	0.279
Length of stay	11.0 (9.0 to 24.5)	10.0 (6.0 to 15.5)	0.498
ICU	3 yes / 32 no	3 yes / 4 no	0.076
WBC	7.1 (5.8 to 9.7)	8.8 (5.5 to 11.6)	0.800
RBC	4.2 (3.9 to 4.6)	4.6 (4.1 to 4.9)	0.273
Hb	13.2 (12.2 to 14.3)	13.9 (13.2 to 14.0)	0.649
PLT	202.0 (152.5 to 298.0)	165.0 (134.0 to 283.0)	0.673
Neutrophils	6.2 (4.2 to 8.1)	6.9 (4.5 to 10.2)	0.673
Lymphocytes	0.9 (0.7 to 1.3)	0.4 (0.4 to 0.7)	0.075
AST	52.0 (31.5 to 80.0)	46.0 (35.0 to 52.0)	0.418
ALT	40.0 (25.5 to 64.5)	23.0 (22.5 to 29.0)	0.021
LDH	382.0 (279.0 to 527.5)	602.0 (400.5 to 680.5)	0.147
GGT	46.0 (34.0 to 96.5)	60.0 (23.0 to 73.0)	0.566
Creatinine	0.9 (0.8 to 1.0)	1.0 (0.9 to 1.1)	0.380
CRP	12.6 (7.9 to 16.1)	10.6 (7.9 to 13.5)	0.716
Ferritin	544.0 (315.5 to 1310.0)	1079.0 (967.5 to 1301.0)	0.224
Fibrinogen	591.0 (446.0 to 650.5)	577.0 (447.0 to 602.0)	0.500
D-Dimer	1383.0 (669.0 to 2261.5)	1137.0 (962.5 to 1733.5)	1.000
Fever*	33 yes / 2 no	7 yes / 0 no	1.000
Cough*	7 yes / 28 no	3 yes / 4 no	0.418
Diarrhea*	5 yes / 30 no	1 yes / 6 no	1.000
Dyspnea*	26 yes / 9 no	6 yes / 1 no	0.871
Comorbidities	17 yes / 18 no	2 yes / 5 no	0.579
Preexisting conditions	15 yes / 20 no	2 yes / 5 no	0.779

ICU, Intensive care unit; WBC, white blood cells (103/mmc); RBC, red blood cells (106/mmc); Hb, haemoglobin (gr/dl); PLT, platelets (103/mmc); AST, Aspartate aminotransferase (UI/l); ALT, Alanine aminotransferase (UI/l); LDH, lactate dehydrogenase (U/l); GGT, Gamma Glutamyl Transferase (UI/l); CRP, C reactive protein (mg/l).

Neutrophils (103/mmc); Lymphocytes (103/mmc); Creatinine (mg/dl); Ferritin (ug/l); Fibrinogen (g/l); D-Dimer (mcg/ml).

*Fever: A measured temperature of 100.4° F (38° C) or greater, or with a history of feeling feverish.; Cough: Continuous cough for more than an hour, or 3 or more coughing episodes in 24 hours; Diarrhea: Loose, watery stools that occur more frequently than usual (at least 3 episodes within a 24-hour period); Dyspnea: Difficult or labored breathing; shortness of breath.

**Fig 1:**
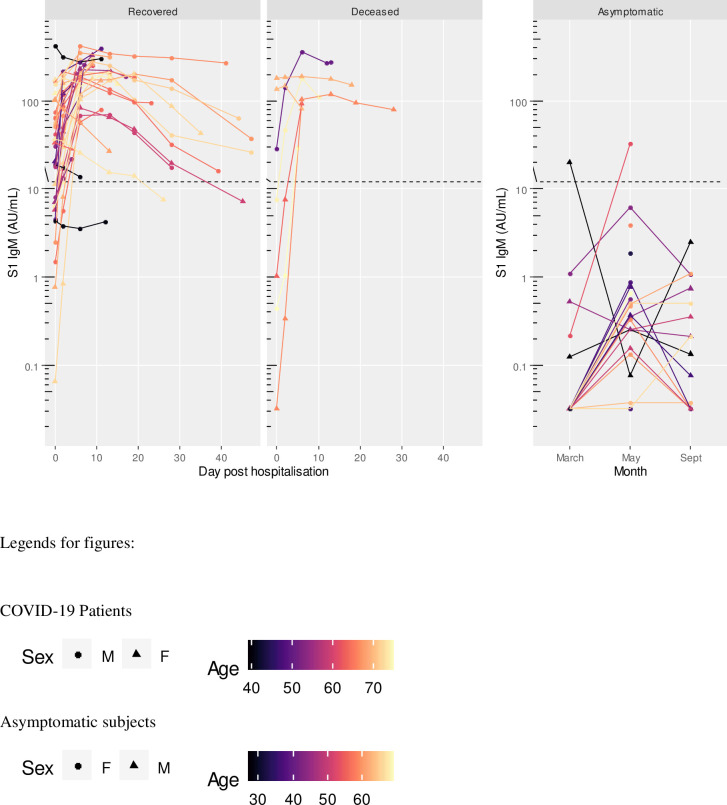
S1 IgM titres in COVID-19 patients (recovered and deceased) and asymptomatic subjects. Black dashed line indicates positivity threshold at 12 U/ml.

**Fig 2:**
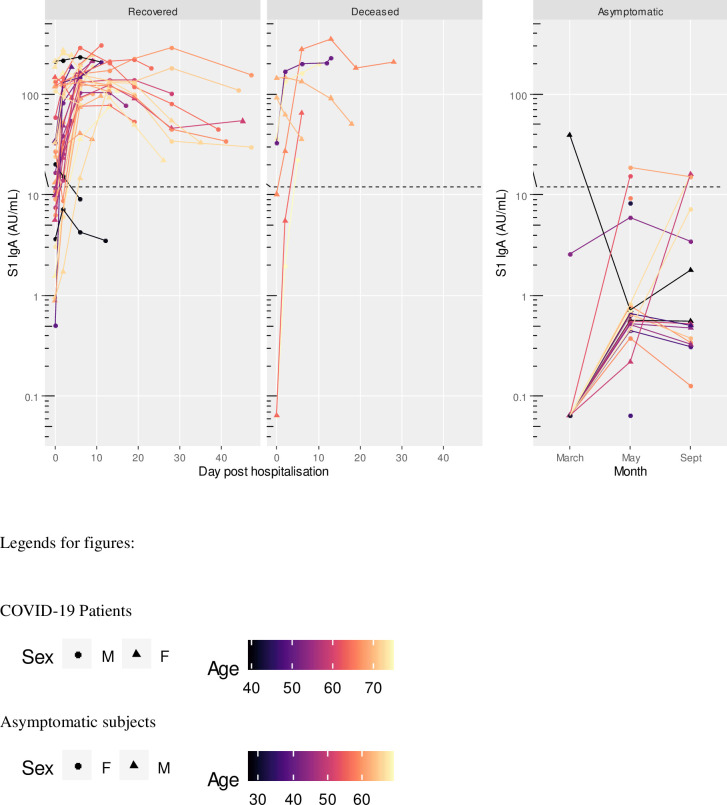
S1 IgA titres in COVID-19 patients (recovered and deceased) and asymptomatic subjects. Black dashed line indicates positivity threshold at 12 U/ml.

**Fig 3:**
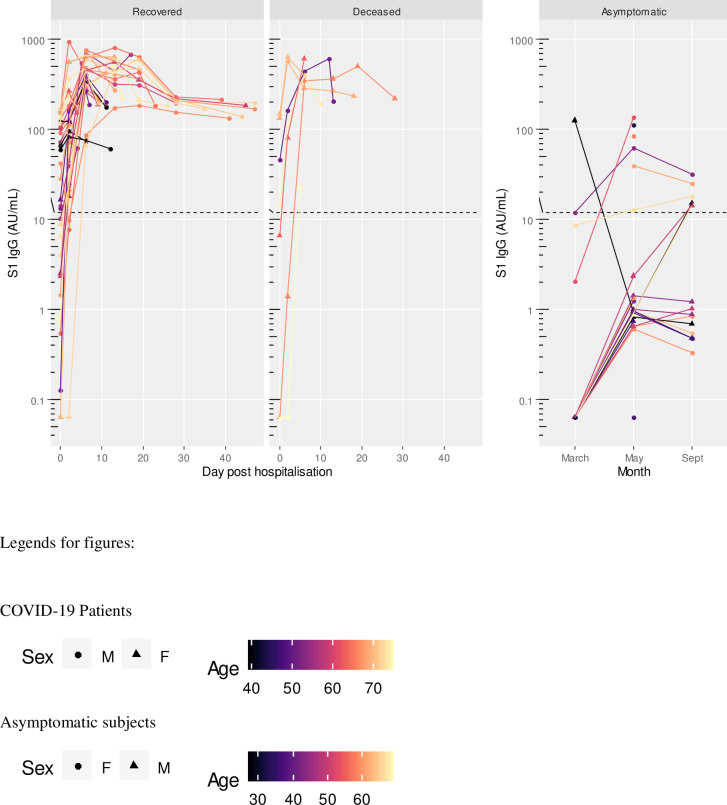
S1 IgG titres in COVID-19 patients (recovered and deceased) and asymptomatic subjects. Black dashed line indicates positivity threshold at 12 U/ml.

**Fig 4:**
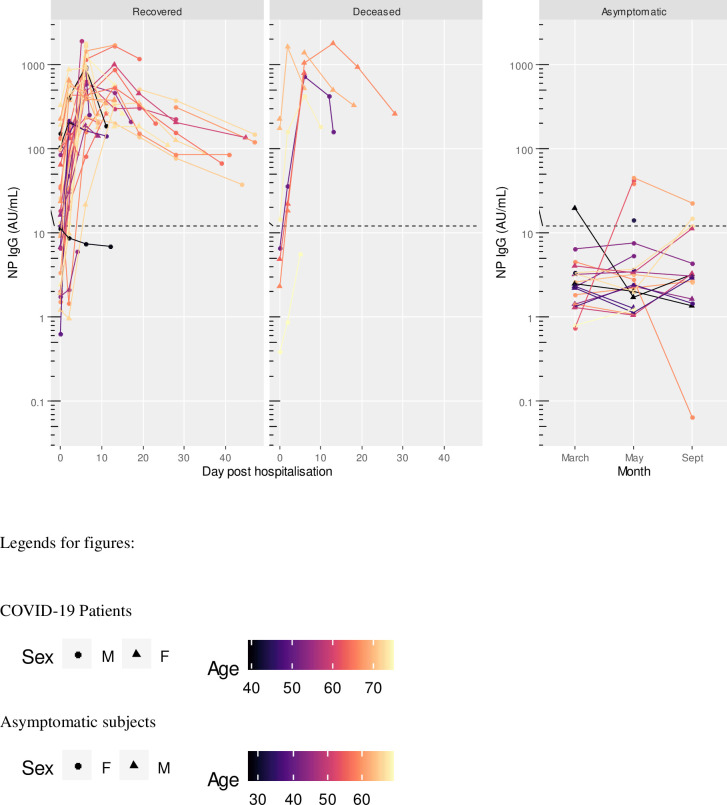
NP IgG titres in COVID-19 patients (recovered and deceased) and asymptomatic subjects. Black dashed line indicates positivity threshold at 12 U/ml.

**Fig 5:**
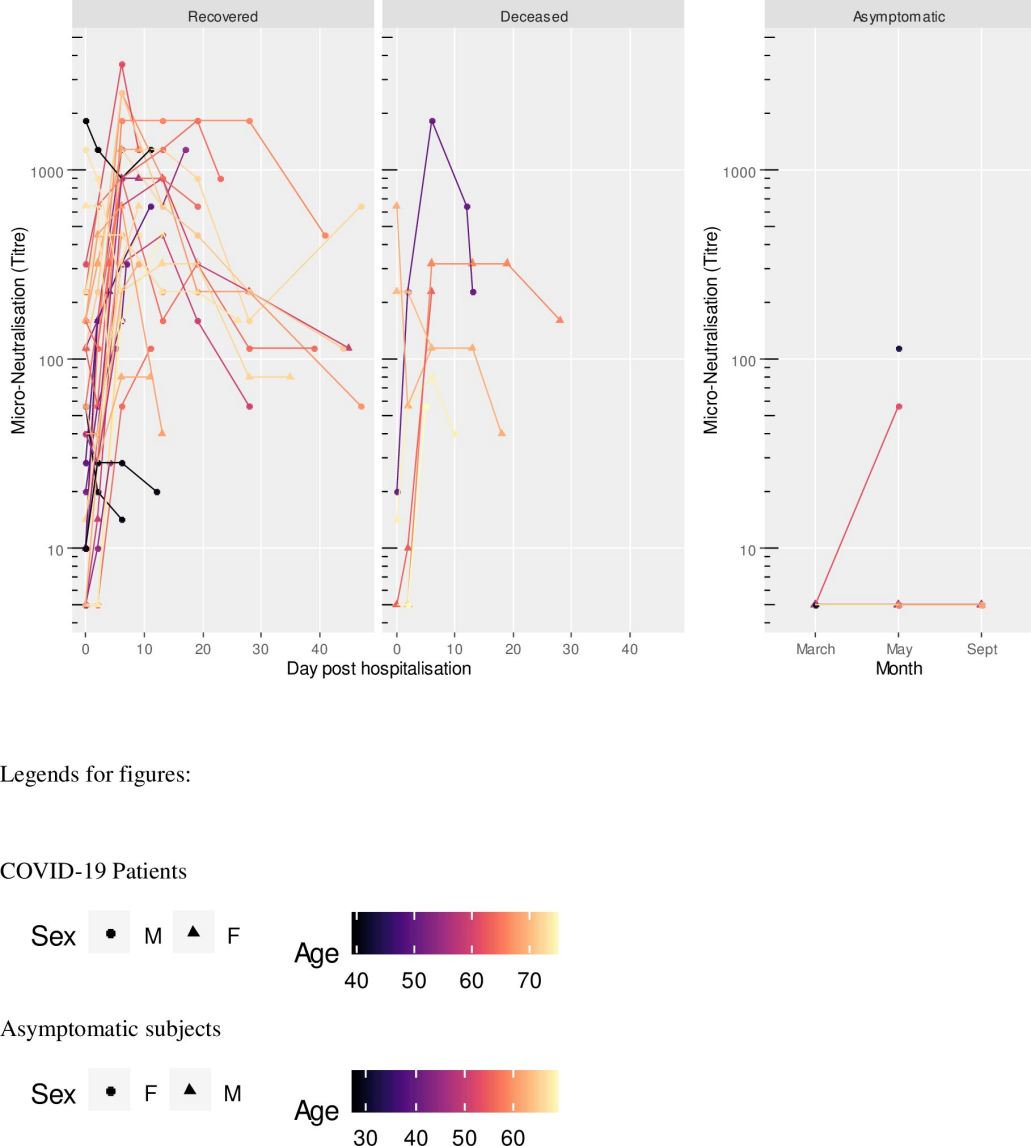
Neutralizing antibody (NAb) titres in COVID-19 patients (recovered and deceased) and asymptomatic subjects.

At hospital admission, 15 patients (35.7%) were negative for S1 IgM, 11 (26.2%) for S1 IgA, 13 (30.9%) for S1 IgG, 15 (35.7%) for NP IgG, and 10 (23.8%) for neutralizing antibodies. Five patients (11.9%) were negative to any antibody assay at the time of admission; of these, 2 died and 3 recovered. Two days after admission, 6 patients (14.3%) were still negative for S1 IgM, 7 (16.7%) for S1 IgA, 4 (9.5%) for S1 IgG, 3 (7.1%) for NP IgG, and 5 (11.9%) for neutralizing antibodies. Two patients (4.8%) were still negative to any antibody assay; of these, 1 died and 1 recovered. At 6 days of sample collection, all subjects except one (97.6%) were positive to all assays (Figs 1–5). The exception was a 40-year-old male patient, positive to S1 IgG at any time point, and borderline for NP IgG only at admission and at day 2. This patient had neutralizing antibody titers less or equal than 40 at any time point. At admission, he had fever and dyspnea with no comorbidities or preexisting conditions and recovered in 12 days.

Two patients, one recovered and one deceased both within 6 days after admission, were both negative to NP IgG at admission and at day 2. Two subjects, both recovered, were positive only for neutralizing antibodies at admission, with titers less or equal to 40. They became positive to all antibodies from day 2 onward (Figs 1–5).

Neutralizing antibodies were found in all patients, with a range from 10 to 5120.

Antibody titers for patients are presented in Table 2. S1 and NP antibodies started increasing at day 2 and again at day 6. A decrease for all antibodies was observed in recovered patients at day 27–30. S1 antibody increase was similar in both recovered and deceased patients, while NP IgG titers were significantly higher in deceased patients at day 6 (p-value 0.044). At baseline, neutralizing antibody titers were 40.8 (95%CI 1.3–1296.4) in recovered patients and 24.4 (95%CI 0.2–3093.8) in those deceased. Already at day 6, neutralizing antibody titers had increased steadily with 427.9 (95% CI 29.0–6321.5) in recovered patients and 226.3 (95% CI 12.1–4228.2) in those deceased. In recovered patients, a plateau of neutralizing antibody titers was observed until day 18–20, followed by a decline at day 27–30. No significant difference was found between the two groups at any time point. However, the comparison at day 27–30 was not possible as only 2 subjects were in the deceased group (Table 2).

**Table 2:** Comparison of immune responses in recovered versus deceased COVID-19 patients.

		Recovered		Deceased			
*Antibody*	*Day*	*N*	*GMT (95% CI)*	*N*	*GMT (95% CI)*	*Ratio D/R*	*P-value*
S1 IgM	Baseline	35	19.6 (0.5 to 718.5)	7	5.0 (0.0 to 12159.3)	0.25 (0.01 to 4.89)	0.308
	Day 2	35	46.9 (3.0 to 732.2)	7	17.4 (0.0 to 9381.0)	0.37 (0.03 to 4.03)	0.356
	Day 6	31	126.7 (16.9 to 951.6)	6	146.4 (34.5 to 621.4)	1.16 (0.61 to 2.17)	0.627
	Day 12–14	18	111.5 (9.7 to 1284.4)	3	177.7 (29.9 to 1057.1)	1.59 (0.70 to 3.63)	0.232
	Day 18–20	12	107.3 (15.6 to 737.9)	2	119.4 (1.7 to 8251.2)	1.00 (1.00 to 1.00)	
	Day 27–30	8	64.4 (5.3 to 788.3)	1	79.2 (0.0 to 0.0)	1.00 (1.00 to 1.00)	
S1 IgA	Baseline	35	17.8 (0.6 to 497.5)	7	6.8 (0.0 to 21390.7)	0.38 (0.02 to 8.03)	0.473
	Day 2	35	42.9 (3.2 to 571.6)	7	34.6 (0.5 to 2510.1)	0.81 (0.16 to 4.13)	0.768
	Day 6	31	96.4 (14.1 to 659.6)	6	118.3 (16.4 to 854.9)	1.23 (0.54 to 2.78)	0.581
	Day 12–14	18	100.2 (15.5 to 646.2)	3	187.0 (10.0 to 3493.1)	1.87 (0.48 to 7.23)	0.249
	Day 18–20	12	108.0 (38.2 to 305.2)	2	95.8 (0.0 to 10573636.2)	1.00 (1.00 to 1.00)	
	Day 27–30	8	79.7 (13.6 to 465.8)	1	206.7 (0.0 to 0.0)	1.00 (1.00 to 1.00)	
S1 IgG	Baseline	35	16.8 (0.1 to 1966.3)	7	6.2 (0.0 to 21044.6)	0.37 (0.02 to 8.18)	0.476
	Day 2	35	66.3 (2.2 to 1956.6)	7	33.5 (0.0 to 147927.2)	0.50 (0.02 to 12.13)	0.623
	Day 6	31	355.8 (90.2 to 1403.1)	6	403.3 (212.0 to 767.1)	1.13 (0.82 to 1.57)	0.436
	Day 12–14	18	397.6 (115.7 to 1366.9)	3	392.4 (65.8 to 2339.2)	0.99 (0.44 to 2.22)	0.964
	Day 18–20	12	408.9 (160.9 to 1038.9)	2	344.5 (0.4 to 335188.3)	1.00 (1.00 to 1.00)	
	Day 27–30	8	202.7 (151.6 to 271.0)	1	220.8 (0.0 to 0.0)	1.00 (1.00 to 1.00)	
NP IgG	Baseline	35	18.1 (0.5 to 647.9)	7	10.8 (0.0 to 2898.3)	0.60 (0.07 to 5.03)	0.588
	Day 2	35	70.3 (2.0 to 2416.5)	7	71.7 (0.1 to 68291.3)	1.02 (0.08 to 13.77)	0.987
	Day 6	31	411.9 (32.3 to 5248.2)	6	756.0 (246.1 to 2323.1)	1.84 (1.02 to 3.32)	0.044
	Day 12–14	18	466.7 (30.1 to 7248.5)	3	725.4 (24.0 to 21884.1)	1.55 (0.34 to 7.06)	0.467
	Day 18–20	12	371.8 (59.2 to 2333.6)	2	550.5 (0.0 to 6914416.1)	1.00 (1.00 to 1.00)	
	Day 27–30	8	171.3 (43.6 to 672.3)	1	262.0 (0.0 to 0.0)	1.00 (1.00 to 1.00)	
NAb	Baseline	35	40.8 (1.3 to 1296.4)	7	24.4 (0.2 to 3093.8)	0.60 (0.09 to 3.81)	0.539
	Day 2	35	75.4 (3.2 to 1754.7)	7	32.8 (0.6 to 1907.7)	0.44 (0.09 to 2.07)	0.255
	Day 6	31	427.9 (29.0 to 6321.5)	6	226.3 (12.1 to 4228.2)	0.53 (0.16 to 1.77)	0.257
	Day 12–14	18	373.3 (27.5 to 5071.8)	3	285.1 (6.7 to 12155.4)	0.76 (0.14 to 4.21)	0.670
	Day 18–20	12	508.0 (79.9 to 3229.0)	2	113.1 (0.0 to 14707762061.6)	1.00 (1.00 to 1.00)	
	Day 27–30	8	190.3 (16.0 to 2267.5)	1	160.0 (0.0 to 0.0)	1.00 (1.00 to 1.00)	

ELISA titres are expressed as U/ml.

NAb, neutralizing antibody.

IgM seroconversion rates were higher in the deceased at day 2 (p-value 0.043), whereas seroconversion rates for the other antibody classes were similar (Table 3).

**Table 3:** Seroconversion in COVID-19 patients according to outcome.

		*Recovered*		*Deceased*		
*Antibody*	*Day*	*SC Yes*	*SC No*	*SC Yes*	*SC No*	*P-value*
S1 IgM	Day 2	6	29	4	3	0.043
	Day 6	19	12	4	2	1.000
	Day 12–14	11	7	2	1	1.000
	Day 18–20	7	5	1	1	1.000
	Day 27–30	4	4	1	0	1.000
S1 IgA	Day 2	8	27	3	4	0.353
	Day 6	20	11	4	2	1.000
	Day 12–14	12	6	2	1	1.000
	Day 18–20	8	4	1	1	1.000
	Day 27–30	5	3	1	0	1.000
S1 IgG	Day 2	11	24	5	2	0.085
	Day 6	23	8	4	2	0.653
	Day 12–14	13	5	2	1	1.000
	Day 18–20	9	3	1	1	0.505
	Day 27–30	4	4	1	0	1.000
NP IgG	Day 2	16	19	6	1	0.096
	Day 6	24	7	5	1	1.000
	Day 12–14	14	4	2	1	1.000
	Day 18–20	9	3	1	1	0.505
	Day 27–30	7	1	1	0	1.000
NAb	Day 2	5	30	1	6	1.000
	Day 6	22	9	4	2	1.000
	Day 12–14	13	5	2	1	1.000
	Day 18–20	9	3	1	1	0.505
	Day 27–30	6	2	1	0	1.000

Seroconversion (SC) was calculated as 4-fold increase in titre compared to baseline.

NAb, neutralizing antibody.

**Fig 6:**
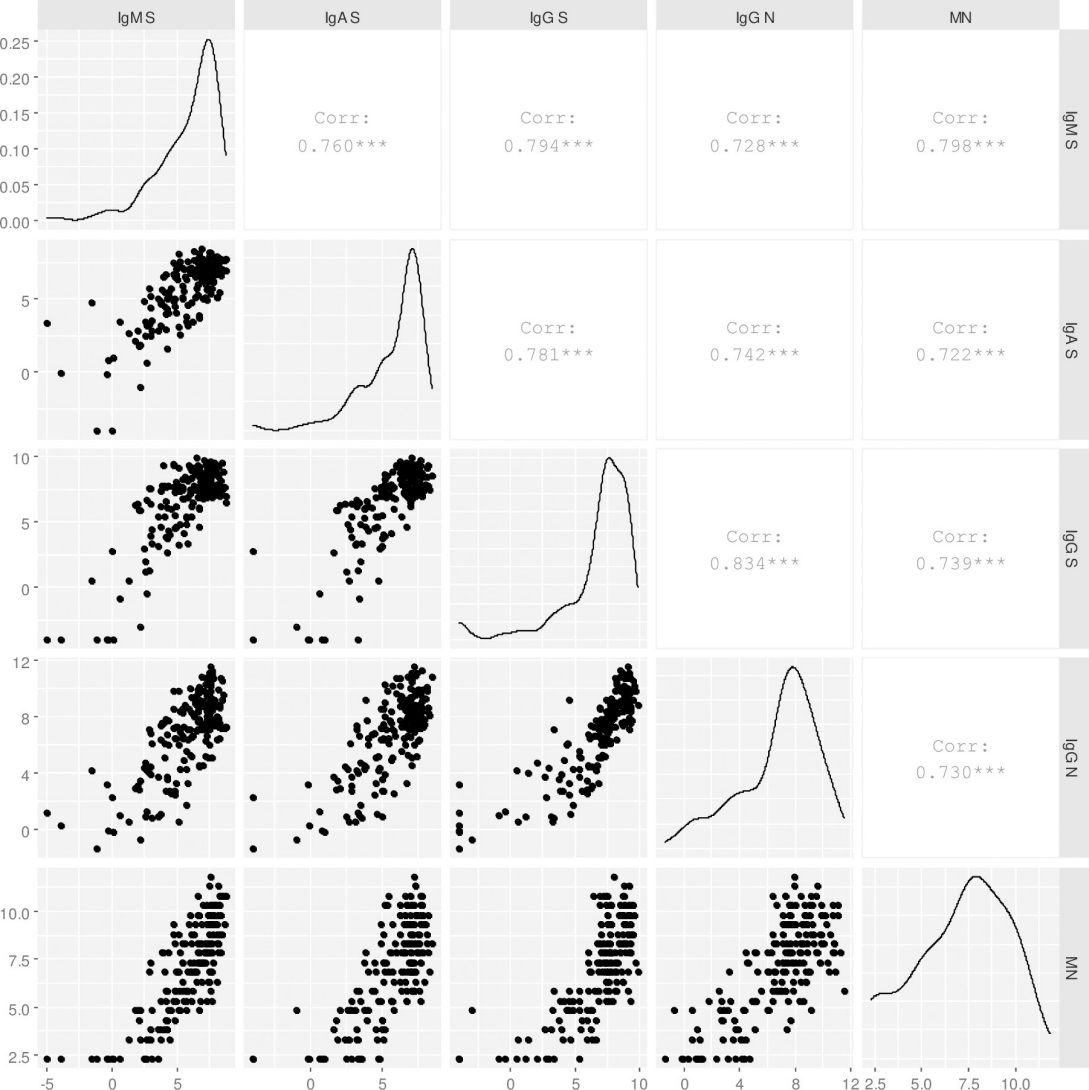
Correlations between antibody for COVID-19 patients. Titres are shown as log-2 transformed.

No significant difference in antibody titers at baseline and by peak antibody level (all 5 assays combined) was found between those who survived and those deceased by using the Cox proportional hazard model. A good correlation was found among all assays as shown in Fig 6. Overall, the level of S1 specific response was well correlated among antibody types (r = 0.781 and r = 0.794, S1 IgG correlating with S1 IgA and S1 IgM, respectively; r = 0.760 S1 IgA correlating with S1 IgM). S1 IgG response was highly correlated with NP IgG (r = 0.834). Neutralizing antibodies well correlated with all ELISA antibodies tested (r = 0.722 with S1 IgA, r = 0.798 with S1 IgM, r = 0.739 with S1 IgG, and r = 0.730 with NP IgG).

### Asymptomatic SARS-CoV-2 infected subjects

Asymptomatic subjects were part of the UNICORN project [33] and serum samples of 25 subjects were kindly provided for the present study. Their median age was 45.0 years (IQR 36.0–60.0), 8 were males and 17 females (Table 4). Twenty-one (21) subjects had the rhino-pharyngeal swab positive to SARS-CoV-2 in March 2020 and a blood draw at the same time, 19 out of the 21 subjects had a second blood draw in May, and 14 of them a third blood draw in September. Four (4) subjects had the rhino-pharyngeal swab positive in May and a blood draw at the same time, of whom 1 subject had a second blood draw in September. Asymptomatic subjects did not receive any medication during the study period that may have interfered with antibody response. Out of 25 asymptomatic subjects, 16 (64.0%) were negative to any antibody at any time point. Nine (9) subjects (36.0%) had at least one detectable antibody type at least at one time point. At the first time point, 6 subjects (24.0%) had positive S1 IgG, of whom 2 also had S1 IgA and NP IgG, 1 subject also had S1 IgM, S1 IgA and NP IgG, and 1 subject had S1 IgA, NP IgG and positive neutralizing antibodies. Three (3) subjects, who were negative at any antibody at the first time point, had antibodies at one of the subsequent time points. One (1) of these subjects was positive to all antibody assays at the second time point including to neutralizing antibodies. The other 2 subjects had S1 IgA, S1 IgG and NP IgG at the third time point. One subject was positive to all ELISA antibodies (S1 IgM, S1 IgA, S1 IgG, and NP IgG) at the first time point, negative at the second time point, and positive again only to S1 IgG at the third time point. Detectable neutralizing antibodies were found only in 2 subjects (8.0%): in one subject at the first and only time point available, and in the other one at the second time point, as the third time point was not available. Both subjects were positive also to S1 IgA, S1 IgG, and NP IgG, and only the first subject was positive to S1 IgM.

**Table 4:** Baseline characteristics of SARS-CoV-2 asymptomatic subjects. Median (IQR).

*Parameter*	*Statistics*
Sex	8 M / 17 F
Smoke	14 never, 5 stopped, 5 active
Age	45.0 (36.0 to 60.0)
BMI	22.8 (21.5 to 24.6)

BMI, Body Mass Index.

Asymptomatic subjects with positive antibody levels in any of the assays had titers well below the level found in patients as shown in Figs 1–5 and Table 5.

**Table 5:** Comparison of baseline immune responses for recovered or deceased versus asymptomatic controls.

Antibody	Recovered/ Asymptomatic	P value	Deceased / Asymptomatic	P value
S1 IgM	286 (111 to 734)	1.42E-15	73 (4 to 1448)	0.0111
S1 IgA	172 (71 to 422)	6.05E-15	65 (3 to 1351)	0.0145
S1 IgG	96 (27 to 338)	4.86e- 9	36 (2 to 798)	0.0295
NP IgG	7.3 (3.7 to 14.3)	3.22e- 7	4.3 (0.5 to 36.3)	0.142
NAb	8.2 (4.6 to 14.6)	1.88e- 8	4.9 (0.8 to 30.5)	0.0785

## Discussion

In this study we primarily evaluated the time course of the antibody response to different antigens of SARS-CoV-2 (IgG, IgM, and IgA against S1, IgG against NP, and neutralizing antibodies) in COVID-19 patients admitted to hospital for interstitial pneumonia during the first epidemic wave in March and April 2020 in Italy. No significant difference in titers was observed in any of the S1 antibody class at any time point between patients who survived and who did not survive.

The only significant difference was the higher S1 IgM seroconversion rate observed in the deceased group that may suggest an early admission to hospital after infection. In other similar studies early antibody response to S1 IgA or IgM or difference in the magnitude of the immune response to SARS-CoV-2 infection was a predictor of disease severity or progression or outcome [34–37]. In this study, IgG antibody titers against NP at day 6 were significantly higher in the deceased group, as reported in other studies where an early response to NP during the first 15 days after disease onset was predictive of fatal outcome [34, 36]. No difference was observed for neutralizing antibodies between the recovered and deceased patients as, on the contrary, reported in other studies where neutralizing antibodies were significantly higher in patients who required ICU or died [36]. One possible explanation for the similar immune response observed in this study in both survived and deceased patients can be the fact that at admission COVID-19 patients had similar clinical and demographic characteristics. In addition, other factors may contribute to the specific immune response against SARS-CoV-2 infection that need to be considered, as the cellular-mediated immunity that may play a role in protection and disease progression.

The kinetics of the antibody response showed an increase starting from day 2 and reaching the peak between days 6 and 18–20. At day 27–30, a decline in titers was observed for any of the antibody classes. In other studies, the antibody kinetic in COVID-19 patients showed the peak at the 4^th^ and 5^th^ week after disease onset, followed by antibody decay starting at the 6^th^ week [37, 38]. This observation differs from our findings most likely due to the fact that our study period started at hospital admission when the severity of the disease was already in an advanced stage.

In this study, almost two-thirds of asymptomatic subjects were negative at any time point for any antibody class, including neutralizing antibodies. Among the few subjects with detectable antibodies, all were positive to S1 IgG, and, none of these subjects was positive to any of the other antibody classes if not positive to S1 IgG. This is difficult to explain. It may be due to the fact that the S protein is deemed the immunodominant antigen of SARS-CoV-2. In accordance with a similar study, antibody titres in asymptomatic subjects were sensibly lower as compared to COVID-19 patients [39]. It has been reported that asymptomatic subjects have a low viral load in the nasopharynges as assessed by RT-PCR, and most likely a lack or a defective viral replication that induces a weak or any antibody response [40]. Although memory B and T cells are likely to be primed in SARS-CoV-2 infected subjects with undetectable antibodies, the question of whether they should be vaccinated is critical now that effective vaccines are available against COVID-19.In this study, a good correlation between S1 and NP protein-based ELISA and the VN assay was observed in COVID-19 patients, although less evident in asymptomatic subjects since only 2 of them had detectable neutralizing antibodies in addition to other ELISA antibody classes. It is acknowledged that antibodies with neutralizing activity should retain enough avidity or have a sufficient concentration or both [41, 42]. In fact, ELISA detects antibody against individual antigens that may not retain neutralizing properties if not in quantity. This may explain the difference in the correlation between ELISA-based assays and neutralizing antibodies in COVID-19 patients and asymptomatic subjects observed in this study.

This study has some limitations. The sample size was small due to the availability of subjects with severe COVID-19 disease who had a sufficient number of blood samples for measuring the time course of antibody for at least one month. This may introduce a bias. However, the ratio between deceased and recovered patients (7 out of 42, 16,6%) falls in the acceptable range (from 5.7% to 30.4%) described in the literature [18, 43]. The retrospective nature of the study and the collection of COVID-19 samples carried out in a single center introduce some limitations. The findings from this study do not allow to predict the kinetics of the antibody decay over time in patients who recovered from COVID-19, and who may be susceptible to reinfection over time, since no follow-up samples after discharge were available.

In conclusion, the results of this study show that COVID-19 patients produce a strong antibody response to SARS-CoV-2 with high correlation between different viral antigens (S1 and NP) and among antibody classes (IgA, IgG, and IgM and neutralizing antibodies). The peak is reached by three weeks from hospital admission followed by a sharp decrease. On the contrary, only few asymptomatic subjects develop antibodies at detectable levels, and significantly lower compared to COVID-19 patients. Currently, no correlates of protection are established for COVID-19. As cases of reinfection are reported [44–47] and since neutralizing antibodies are rarely produced in asymptomatic subjects, the findings of this study support the current recommendation to vaccinate subjects with a previous SARS-CoV-2 infection as well as those who recovered from COVID-1.

## References

[1] World Health Organization. WHO Director-General’s opening remarks at the media briefing on COVID-19–11 March 2020. 2020; Available from: https://www.who.int/dg/speeches/detail/who-director-general-s-opening-remarks-at-the-media-briefing-on-covid-19—11-march-2020.

[2] Lu R., Zhao X., Li J., Niu P., Yang B., Wu H., et al., Genomic characterisation and epidemiology of 2019 novel coronavirus: implications for virus origins and receptor binding. Lancet, 2020. 395(10224): p. 565–574. doi: 10.1016/S0140-6736(20)30251-8 32007145 PMC7159086

[3] Tay M.Z., Poh C.M., Renia L., MacAry P.A., and Ng L.F.P., The trinity of COVID-19: immunity, inflammation and intervention. Nat Rev Immunol, 2020. 20(6): p. 363–374. doi: 10.1038/s41577-020-0311-8 32346093 PMC7187672

[4] Amanat F. and Krammer F., SARS-CoV-2 Vaccines: Status Report. Immunity, 2020. 52(4): p. 583–589. doi: 10.1016/j.immuni.2020.03.007 32259480 PMC7136867

[5] Chan J.F., Yuan S., Kok K.H., To K.K., Chu H., Yang J., et al., A familial cluster of pneumonia associated with the 2019 novel coronavirus indicating person-to-person transmission: a study of a family cluster. Lancet, 2020. 395(10223): p. 514–523. doi: 10.1016/S0140-6736(20)30154-9 31986261 PMC7159286

[6] Zhu N., Zhang D., Wang W., Li X., Yang B., Song J., et al. China Novel Coronavirus, and T. Research, A Novel Coronavirus from Patients with Pneumonia in China, 2019. N Engl J Med, 2020. 382(8): p. 727–733. doi: 10.1056/NEJMoa2001017 31978945 PMC7092803

[7] World Health Organization. MERS SITUATION UPDATE. 2019 02/16/2021]; Available from: https://applications.emro.who.int/docs/EMRPUB-CSR-241-2019-EN.pdf?ua=1&ua=1&ua=1.

[8] Du L.Y., He Y.X., Zhou Y.S., Liu S.W., Zheng B.J., and Jiang S.B., The spike protein of SARS-CoV—a target for vaccine and therapeutic development. Nature Reviews Microbiology, 2009. 7(3): p. 226–236. doi: 10.1038/nrmicro2090 19198616 PMC2750777

[9] Huang C., Wang Y., Li X., Ren L., Zhao J., Hu Y., et al., Clinical features of patients infected with 2019 novel coronavirus in Wuhan, China. Lancet, 2020. 395(10223): p. 497–506. doi: 10.1016/S0140-6736(20)30183-5 31986264 PMC7159299

[10] Jiang S., Hillyer C., and Du L., Neutralizing Antibodies against SARS-CoV-2 and Other Human Coronaviruses: (Trends in Immunology 41, 355–359; 2020). Trends Immunol, 2020. 41(6): p. 545. doi: 10.1016/j.it.2020.04.008 32249063 PMC7129017

[11] Du L., Yang Y., Zhou Y., Lu L., Li F., and Jiang S., MERS-CoV spike protein: a key target for antivirals. Expert Opin Ther Targets, 2017. 21(2): p. 131–143. doi: 10.1080/14728222.2017.1271415 27936982 PMC5457961

[12] Wajnberg A., Amanat F., Firpo A., Altman D.R., Bailey M.J., Mansour M., et al, Robust neutralizing antibodies to SARS-CoV-2 infection persist for months. Science, 2020. doi: 10.1126/science.abd7728 33115920 PMC7810037

[13] Seow J., Graham C., Merrick B., Acors S., Pickering S., Steel K.J.A., et al., Longitudinal observation and decline of neutralizing antibody responses in the three months following SARS-CoV-2 infection in humans. Nat Microbiol, 2020. doi: 10.1038/s41564-020-00813-8 33106674 PMC7610833

[14] Amanat F., Stadlbauer D., Strohmeier S., Nguyen T.H.O., Chromikova V., McMahon M., et al., A serological assay to detect SARS-CoV-2 seroconversion in humans. Nat Med, 2020. 26(7): p. 1033–1036. doi: 10.1038/s41591-020-0913-5 32398876 PMC8183627

[15] To K.K., Tsang O.T., Leung W.S., Tam A.R., Wu T.C., Lung D.C., et al., Temporal profiles of viral load in posterior oropharyngeal saliva samples and serum antibody responses during infection by SARS-CoV-2: an observational cohort study. Lancet Infect Dis, 2020. doi: 10.1016/S1473-3099(20)30196-1 32213337 PMC7158907

[16] Haveri A., Smura T., Kuivanen S., Osterlund P., Hepojoki J., Ikonen N., et al., Serological and molecular findings during SARS-CoV-2 infection: the first case study in Finland, January to February 2020. Euro Surveill, 2020. 25(11). doi: 10.2807/1560-7917.ES.2020.25.11.2000266 32209163 PMC7096774

[17] Long Q.X., Liu B.Z., Deng H.J., Wu G.C., Deng K., Chen Y.K., et al., Antibody responses to SARS-CoV-2 in patients with COVID-19. Nat Med, 2020. 26(6): p. 845–848. doi: 10.1038/s41591-020-0897-1 32350462

[18] Roltgen K., Powell A.E., Wirz O.F., Stevens B.A., Hogan C.A., Najeeb J., et al., Defining the features and duration of antibody responses to SARS-CoV-2 infection associated with disease severity and outcome. Sci Immunol, 2020. 5(54). doi: 10.1126/sciimmunol.abe0240 33288645 PMC7857392

[19] Heald-Sargent T. and Gallagher T., Ready, set, fuse! The coronavirus spike protein and acquisition of fusion competence. Viruses, 2012. 4(4): p. 557–80. doi: 10.3390/v4040557 22590686 PMC3347323

[20] Manenti A., Maggetti M., Casa E., Martinuzzi D., Torelli A., Trombetta C.M., et al., Evaluation of SARS-CoV-2 neutralizing antibodies using a CPE-based colorimetric live virus micro-neutralization assay in human serum samples. J Med Virol, 2020. 92(10): p. 2096–2104. doi: 10.1002/jmv.25986 32383254 PMC7267461

[21] WHO, Manual for the laboratory diagnosis and virological surveillance of influenza. 2011. p. 1–153.

[22] Young B.E., Ong S.W.X., Kalimuddin S., Low J.G., Tan S.Y., Loh J., et al., Epidemiologic Features and Clinical Course of Patients Infected With SARS-CoV-2 in Singapore. JAMA, 2020. 323(15): p. 1488–1494. doi: 10.1001/jama.2020.3204 32125362 PMC7054855

[23] World Health Organization. WHO Coronavirus Disease (COVID-19) Dashboard. 2021 12/13/2020]; Available from: https://covid19.who.int/.

[24] Food and Agriculture Organization of the United Nations. Impact of COVID-19 on people’s livelihoods, their health and our food systems. 2020 02/1672021]; Available from: http://www.fao.org/news/story/en/item/1313598/icode/.

[25] Apolone G., Montomoli E., Manenti A., Boeri M., Sabia F., Hyseni I., et al., Unexpected detection of SARS-CoV-2 antibodies in the prepandemic period in Italy. Tumori, 2020: p. 300891620974755. doi: 10.1177/0300891620974755 33176598 PMC8529295

[26] Deslandes A., Berti V., Tandjaoui-Lambotte Y., Alloui C., Carbonnelle E., Zahar J.R., et al., SARS-CoV-2 was already spreading in France in late December 2019. Int J Antimicrob Agents, 2020. 55(6): p. 106006. doi: 10.1016/j.ijantimicag.2020.106006 32371096 PMC7196402

[27] Amendola A., Bianchi S., Gori M., Colzani D., Canuti M., Borghi E., et al., Evidence of SARS-CoV-2 RNA in an Oropharyngeal Swab Specimen, Milan, Italy, Early December 2019. Emerg Infect Dis, 2021. 27(2): p. 648–650. doi: 10.3201/eid2702.204632 33292923 PMC7853584

[28] Gianotti R., Barberis M., Fellegara G., Galvan-Casas C., and Gianotti E., COVID-19-related dermatosis in November 2019: could this case be Italy’s patient zero? Br J Dermatol, 2021. doi: 10.1111/bjd.19804 33410129 PMC9619443

[29] Istituto Superiore di Sanità, Caratteristiche dei pazienti deceduti positivi all’infezione da SARS-CoV-2 in Italia. 2020.

[30] Onder G., Rezza G., and Brusaferro S., Case-Fatality Rate and Characteristics of Patients Dying in Relation to COVID-19 in Italy. JAMA, 2020. 323(18): p. 1775–1776. doi: 10.1001/jama.2020.4683 32203977

[31] Istituto Superiore di Sanità, EPIDEMIA COVID-19. Aggiornamento nazionale 2 Aprile 2020. 2020.

[32] Lipsitch M., Swerdlow D.L., and Finelli L., Defining the Epidemiology of Covid-19—Studies Needed. N Engl J Med, 2020. 382(13): p. 1194–1196. doi: 10.1056/NEJMp2002125 32074416

[33] Milani G.P., Dioni L., Favero C., Cantone L., Macchi C., Delbue S., et al., Serological follow-up of SARS-CoV-2 asymptomatic subjects. Sci Rep, 2020. 10(1): p. 20048. doi: 10.1038/s41598-020-77125-8 33208819 PMC7674414

[34] Atyeo C., Fischinger S., Zohar T., Slein M.D., Burke J., Loos C., et al., Distinct Early Serological Signatures Track with SARS-CoV-2 Survival. Immunity, 2020. 53(3): p. 524–+. doi: 10.1016/j.immuni.2020.07.020 32783920 PMC7392190

[35] Wang Y.Q., Zhang L., Sang L., Ye F., Ruan S.C., Zhong B., et al., Kinetics of viral load and antibody response in relation to COVID-19 severity. Journal of Clinical Investigation, 2020. 130(10): p. 5235–5244. doi: 10.1172/JCI138759 32634129 PMC7524490

[36] Hashem A.M., Algaissi A., Almahboub S.A., Alfaleh M.A., Abujamel T.S., Alamri S.S., et al., Early Humoral Response Correlates with Disease Severity and Outcomes in COVID-19 Patients. Viruses, 2020. 12(12). doi: 10.3390/v12121390 33291713 PMC7761967

[37] Chen Y., Tong X., Li Y., Gu B., Yan J., Liu Y., et al., A comprehensive, longitudinal analysis of humoral responses specific to four recombinant antigens of SARS-CoV-2 in severe and non-severe COVID-19 patients. PLoS Pathog, 2020. 16(9): p. e1008796. doi: 10.1371/journal.ppat.1008796 32913364 PMC7482996

[38] Okba N.M.A., Muller M.A., Li W., Wang C., GeurtsvanKessel C.H., Corman V.M., et al., Severe Acute Respiratory Syndrome Coronavirus 2-Specific Antibody Responses in Coronavirus Disease Patients. Emerg Infect Dis, 2020. 26(7): p. 1478–1488. doi: 10.3201/eid2607.200841 32267220 PMC7323511

[39] Algaissi A., Alfaleh M.A., Hala S., Abujamel T.S., Alamri S.S., Almahboub S.A., et al., SARS-CoV-2 S1 and N-based serological assays reveal rapid seroconversion and induction of specific antibody response in COVID-19 patients. Sci Rep, 2020. 10(1): p. 16561. doi: 10.1038/s41598-020-73491-5 33024213 PMC7538990

[40] Röltgen K., Powell A.E., Wirz O.F., Stevens B.A., Hogan C.A., Najeeb J., et al., Defining the features and duration of antibody responses to SARS-CoV-2 infection associated with disease severity and outcome. ScienceImmunology, 2020, p. 1–19. doi: 10.1126/sciimmunol.abe0240 33288645 PMC7857392

[41] Piccoli L., Park Y.J., Tortorici M.A., Czudnochowski N., Walls A.C., Beltramello M., et al., Mapping Neutralizing and Immunodominant Sites on the SARS-CoV-2 Spike Receptor-Binding Domain by Structure-Guided High-Resolution Serology. Cell, 2020. 183(4): p. 1024–+. doi: 10.1016/j.cell.2020.09.037 32991844 PMC7494283

[42] Burton D.R., Williamson R.A., and Parren P.W., Antibody and virus: binding and neutralization. Virology, 2000. 270(1): p. 1–3. doi: 10.1006/viro.2000.0239 10772973

[43] Yao T., Gao Y., Cui Q., Peng B., Chen Y., Li J., et al., Clinical characteristics of a group of deaths with COVID-19 pneumonia in Wuhan, China: a retrospective case series. BMC Infect Dis, 2020. 20(1): p. 695. doi: 10.1186/s12879-020-05423-7 32962639 PMC7506806

[44] Iwasaki A., What reinfections mean for COVID-19. Lancet, 2020. 21: p. 3–5. doi: 10.1016/S1473-3099(20)30783-0 33058796 PMC7550040

[45] Tillett R.L., Sevinsky J.R., Hartley P.D., Kerwin H., Crawford N., Gorzalski A., et al., Genomic evidence for reinfection with SARS-CoV-2: a case study. Lancet Infect Dis, 2021. 21(1): p. 52–58. doi: 10.1016/S1473-3099(20)30764-7 33058797 PMC7550103

[46] Hansen C.H., Michlmayr D., and et al., Assessment of protection against reinfection with SARS-CoV-2 among 4 million PCR-tested individuals in Denmark in 2020: a population-level observational study. Lancet, 2021. 397: p. 1204–12. doi: 10.1016/S0140-6736(21)00575-4 33743221 PMC7969130

[47] Abu-Raddad L.J. and Chemaitelly H., SARS-CoV-2 reinfection in a cohort of 43,000 antibody-positive individuals followed for up to 35 weeks. MedRxiv, 2021.

